# Supplementary material for: Interpretable machine learning for predicting delays in seeking abortion among reproductive-aged women in Ethiopia: A study using EDHS 2016 data
Source: PLOS Digit Health. 2026 Mar 13;5(3):e0001288. doi: 10.1371/journal.pdig.0001288 (PMC12987417; doi:10.1371/journal.pdig.0001288)
Supplement: S1 Text — (DOCX) [file pdig.0001288.s001.docx]

**Supplementary files for Interpretable Machine Learning for Predicting** **Delays in Seeking Abortion Among Reproductive-Aged Women in Ethiopia: A Study Using EDHS 2016 Data.**

Table A in S1 Text: Socio-demographic characteristics for Delays in Seeking Abortion Among Reproductive-Aged Women in Ethiopia.

|  | | **Delayed Abortion Seeking** | | | |
| --- | --- | --- | --- | --- | --- |
|  |  | **Not Delayed** | | **Delayed** | |
|  |  | **Frequency** | **%** | **Frequency** | **%** |
| Age of the women | 15-19 | 8 | 0.9 | 11 | 1.0 |
|  | 20-24 | 63 | 6.8 | 71 | 6.4 |
|  | 25-29 | 217 | 23.3 | 222 | 20.0 |
|  | 30-34 | 248 | 26.6 | 203 | 18.3 |
|  | 35-39 | 254 | 27.2 | 275 | 24.8 |
|  | 40-44 | 94 | 10.1 | 207 | 18.7 |
|  | 45-49 | 49 | 5.3 | 120 | 10.8 |
| Region | Tigray | 103 | 11.0 | 137 | 12.4 |
|  | Afar | 108 | 11.6 | 124 | 11.2 |
|  | Amhara | 32 | 3.4 | 159 | 14.3 |
|  | Oromia | 190 | 20.4 | 180 | 16.2 |
|  | Somali | 97 | 10.4 | 207 | 18.7 |
|  | Benishangul | 48 | 5.1 | 62 | 5.6 |
|  | Snnpr | 116 | 12.4 | 80 | 7.2 |
|  | Gambela | 27 | 2.9 | 38 | 3.4 |
|  | Harari | 18 | 1.9 | 37 | 3.3 |
|  | Addis Adaba | 108 | 11.6 | 27 | 2.4 |
|  | Dire Dawa | 86 | 9.2 | 58 | 5.2 |
| Place of residence | Urban | 253 | 27.1 | 149 | 13.4 |
|  | Rural | 680 | 72.9 | 960 | 86.6 |
| Highest educational level | No Education | 583 | 62.5 | 819 | 73.9 |
|  | Primary | 232 | 24.9 | 244 | 22.0 |
|  | Secondary | 77 | 8.3 | 31 | 2.8 |
|  | Higher | 41 | 4.4 | 15 | 1.4 |
| Chews/Smokes Tobacco | No | 933 | 100.0 | 1101 | 99.3 |
|  | Yes | 0 | 0.0 | 8 | 0.7 |
| Marital Status | Never In Union | 4 | 0.4 | 5 | 0.5 |
|  | Married | 867 | 92.9 | 1060 | 95.6 |
|  | Living With Partner | 7 | 0.8 | 6 | 0.5 |
|  | Widowed | 12 | 1.3 | 6 | 0.5 |
|  | Divorced | 34 | 3.6 | 20 | 1.8 |
|  | No Longer Living Together/Separated | 9 | 1.0 | 12 | 1.1 |
| Decision Maker For Not Using Contraception | Mainly Respondent | 130 | 24.2 | 224 | 30.8 |
|  | Mainly Husband, Partner | 56 | 10.4 | 66 | 9.1 |
|  | Joint Decision | 345 | 64.1 | 412 | 56.6 |
|  | Other | 7 | 1.3 | 26 | 3.6 |
| Husband/Partner's Education Level | No Education | 368 | 42.1 | 535 | 50.2 |
|  | Primary | 335 | 38.3 | 368 | 34.5 |
|  | Secondary | 94 | 10.8 | 87 | 8.2 |
|  | Higher | 77 | 8.8 | 56 | 5.3 |
|  | Don't Know | 0 | 0.0 | 20 | 1.9 |
| Husband/Partner's Occupation | Did Not Work | 97 | 11.1 | 134 | 12.6 |
|  | Professional/Technical/Managerial | 77 | 8.8 | 86 | 8.1 |
|  | Clerical | 11 | 1.3 | 3 | 0.3 |
|  | Sales | 69 | 7.9 | 44 | 4.1 |
|  | Agricultural - Self Employed | 0 | 0.0 | 0 | 0.0 |
|  | Agricultural - Employee | 421 | 48.2 | 595 | 55.8 |
|  | Household And Domestic | 0 | 0.0 | 0 | 0.0 |
|  | Services | 41 | 4.7 | 36 | 3.4 |
|  | Skilled Manual | 55 | 6.3 | 87 | 8.2 |
|  | Unskilled Manual | 59 | 6.8 | 39 | 3.7 |
|  | Others | 30 | 3.4 | 42 | 3.9 |
|  | Don't Know | 14 | 1.6 | 0 | 0.0 |
| Respondent's Occupation | Not Working | 471 | 50.5 | 580 | 52.3 |
|  | Professional/Technical/Managerial | 25 | 2.7 | 20 | 1.8 |
|  | Clerical | 6 | 0.6 | 8 | 0.7 |
|  | Sales | 176 | 18.9 | 124 | 11.2 |
|  | Agricultural - Self Employed | 0 | 0.0 | 0 | 0.0 |
|  | Agricultural - Employee | 187 | 20.0 | 247 | 22.3 |
|  | Household And Domestic | 0 | 0.0 | 0 | 0.0 |
|  | Services | 16 | 1.7 | 24 | 2.2 |
|  | Skilled Manual | 26 | 2.8 | 52 | 4.7 |
|  | Unskilled Manual | 17 | 1.8 | 26 | 2.3 |
|  | Others | 9 | 1.0 | 28 | 2.5 |
|  | Don't Know | 0 | 0.0 | 0 | 0.0 |
| Alcohol Drinking | No | 682 | 73.1 | 758 | 68.3 |
|  | Yes | 251 | 26.9 | 351 | 31.7 |
| Media Exposure | No | 493 | 52.8 | 726 | 65.5 |
|  | Yes | 440 | 47.2 | 383 | 34.5 |
| Number of Household members | 1-5 | 350 | 37.5 | 338 | 30.5 |
|  | 6-12 | 566 | 60.7 | 764 | 68.9 |
|  | >13 | 17 | 1.8 | 7 | 0.6 |
| Number of Under five children | 1-3 | 933 | 100.0 | 1097 | 98.9 |
|  | >3 | 0 | 0.0 | 12 | 1.1 |
| Wealth Index | Poor | 416 | 44.6 | 613 | 55.3 |
|  | Middle | 127 | 13.6 | 148 | 13.3 |
|  | Rich | 390 | 41.8 | 348 | 31.4 |
| Total children ever born | 1-5 | 500 | 53.6 | 520 | 46.9 |
|  | >6 | 433 | 46.4 | 589 | 53.1 |


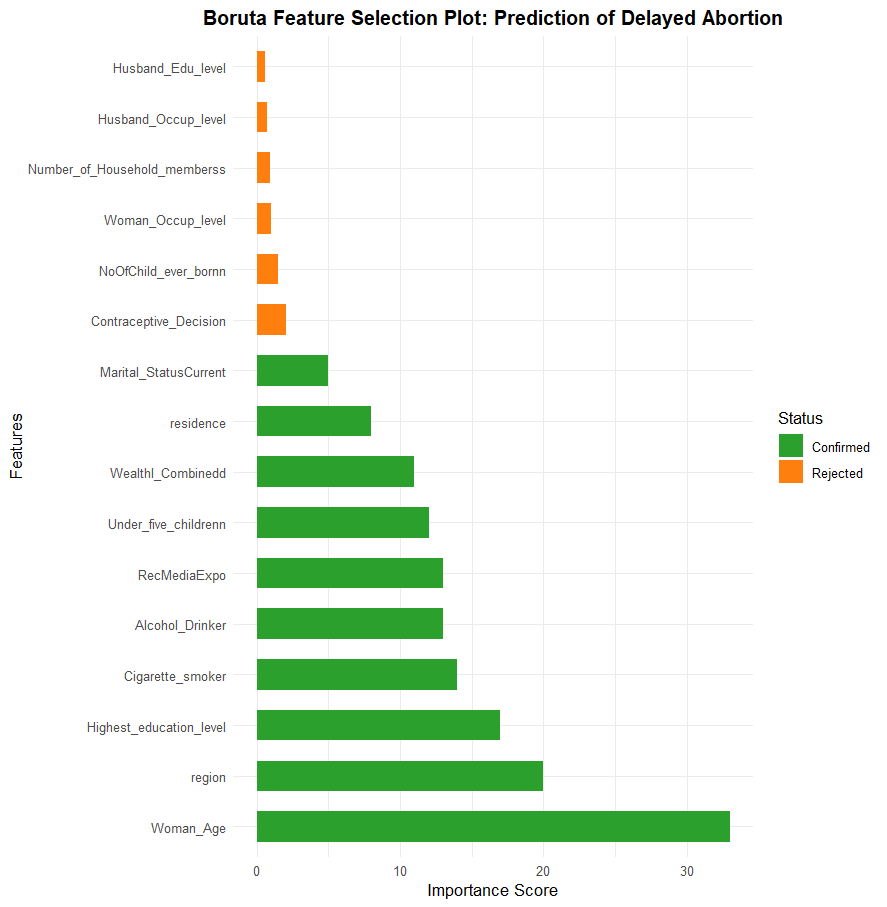


Fig A in S1 Text: Feature Selection with Boruta for prediction of Delayed Abortion


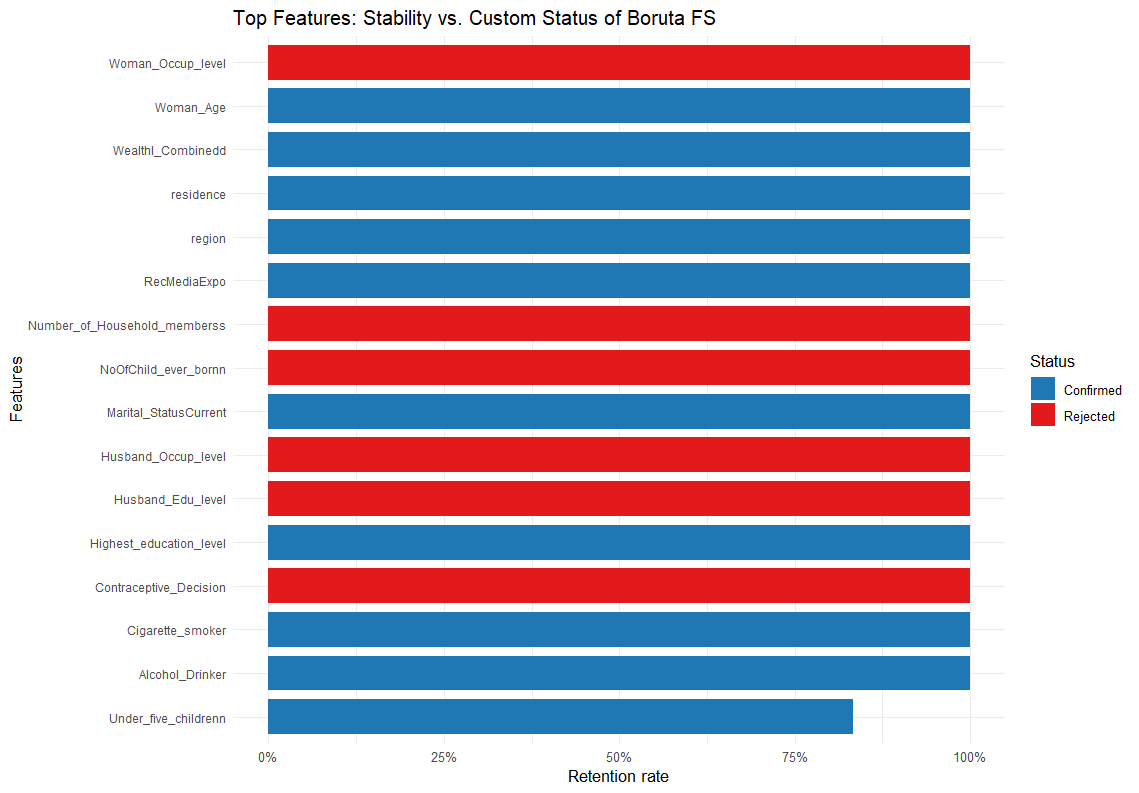


Fig B in S1 Text: Stability of Boruta‑selected features across 30 subsampled training sets. Bars show the proportion of subsamples in which each feature was classified as “Confirmed” or “Rejected”. Stable features (≥80% retention) indicate high reproducibility of Boruta feature selection.

Table B in S1 Text: Hyperparameter Tuning for Machine Learning Models: Default and Optimal Values

| **ML Model** | **Hyperparameters** | **Default Values** | **Optimal Values** |
| --- | --- | --- | --- |
| **XGBoost (XGB)** | max_depth | 6 | 14 |
|  | min_child_weight | 1 | 0.8 |
|  | gamma | 0 | 1 |
|  | eta (learning rate) | 0.3 | 0.1 |
|  | colsample_bytree | 1 | 1 |
|  | objective | reg:squarederror | binary:logistic |
|  | eval_metric | rmse | auc |
|  | nrounds | 100 | 4000 |
|  | early_stopping_rounds | - (not default) | 20 (early stopping) |
| **Random Forest (RF)** | ntrees | 100 | 50 |
|  | max_depth | 20 | 20 |
|  | min_rows | 10 | 1 |
|  | sample_rate | 1.0 | 0.8 |
|  | nfolds | 0 | 10 (cross-validation) |
|  | fold_assignment | - | Stratified |
|  | stopping_rounds | - | 50 |
|  | stopping_metric | AUC | RMSE |
| **Support Vector Machine (SVM)** | method | svmRadial | svmRadial |
|  | preProcess | none | center, scale |
|  | classProbs | FALSE | TRUE |
|  | summaryFunction | - | twoClassSummary (for AUC) |
|  | tuneLength | 3 | 10 |
| **Naive Bayes (NB)** | method | naive_bayes | naive_bayes |
|  | trControl | none | repeatedcv (10 folds, 5 repeats) |
|  | metric | Accuracy | Accuracy |
| **K-Nearest Neighbors (KNN)** | k | - | Values from 1 to nrow(DA_train)/3 (20 values tested) |
|  | method | knn | knn |
|  | trControl | none | repeatedcv (10 folds, 5 repeats) |
|  | classProbs | FALSE | TRUE |
|  | summaryFunction | - | twoClassSummary (for AUC) |
| **Logistic Regression (LR)** | method | glm | glm (binomial family) |
|  | family | gaussian | binomial |
|  | trControl | none | repeatedcv (10 folds, 5 repeats) |
| **LightGBM (LGBM)** | objective | regression | binary |
|  | metric | rmse | binary_logloss |
|  | learning_rate | 0.05 | 0.1 |
|  | num_leaves | 31 | 31 |
|  | max_depth | -1 | -1 |
|  | min_data_in_leaf | 20 | 20 |
|  | nrounds | 100 | 100 |
|  | early_stopping_rounds | - | 10 |
| **Gradient Boosting Machine (GBM)** | learning_rate | 0.1 | Values tested: 0.3, 0.1, 0.05, 0.01, 0.005 |
|  | interaction.depth | 3 | 3 |
|  | n.trees | 1000 | 1000 |
|  | cv.folds | 10 | 5 (cross-validation) |
|  | n.minobsinnode | 10 | 10 |


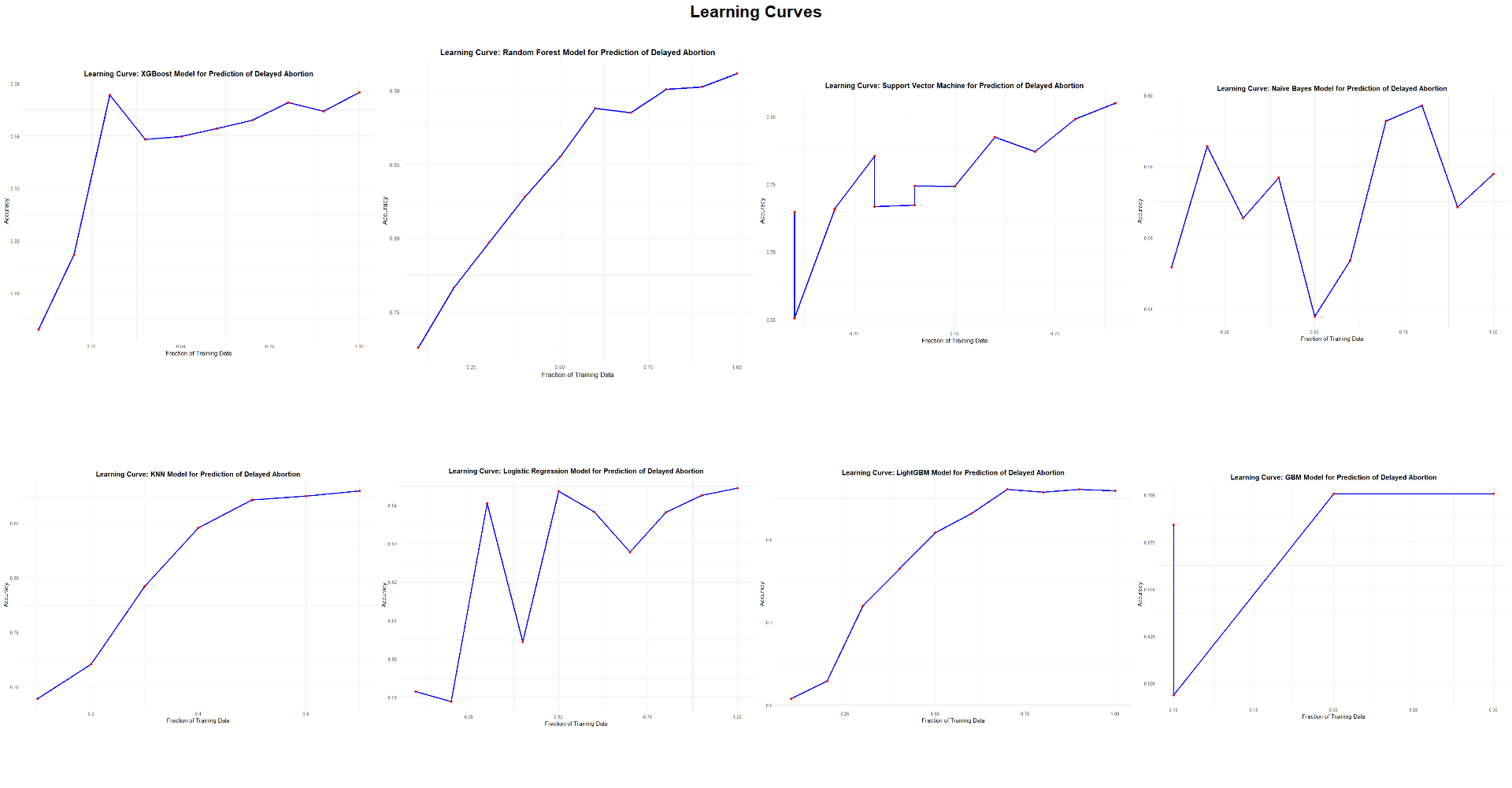


Fig C in S1 Text: The Learning Curve of the Machine Learning Models implemented in the study. Order [XGB, RF, SVM, NB, KNN, LG, LGBM, GBM]
