# Supplementary material for: Interpretable machine learning for predicting delays in seeking abortion among reproductive-aged women in Ethiopia: A study using EDHS 2016 data
Source: PLOS Digit Health. 2026 Mar 13;5(3):e0001288. doi: 10.1371/journal.pdig.0001288 (PMC12987417; doi:10.1371/journal.pdig.0001288)
Supplement: Attachment — Submitted filename: Response to Reviewers R1.pdf [file pdig.0001288.s003.pdf]

Point by Point Response

We would like to express our sincere gratitude to the editor and reviewers for their insightful and constructive comments on our manuscript titled '*Interpretable Machine Learning for Predicting Delays in Seeking Abortion Among Reproductive-Aged Women in Ethiopia: A Study Using EDHS 2016 Data*' We have carefully considered each suggestion and made the necessary revisions to improve the clarity, depth, and overall quality of our manuscript. In the following sections, we provide a detailed point-by-point response to each comment, outlining the changes made in response to the feedback received. We have also processed our figure files using the PACE (Preflight Analysis and Conversion Engine) tool, as instructed, to ensure they meet PLOS's formatting standards.

We believe these revisions have significantly enhanced the rigor and impact of our work, and we are confident that the manuscript is now stronger as a result of this valuable input. All line numbers refers to the revised manuscript with yellow highlights.

Review Comments to the Author

Reviewer #1:

Thank you to look over my insight full expertise on this research work entitled "Interpretable Machine Learning for Predicting Delays in Seeking Abortion Among Reproductive-Aged Women in Ethiopia: : A Study Using EDHS 2016 Data" and I got this article is very interesting and thought full and identifying deferent Hedin factors that why mothers delay in Seeking Abortion service. The research work is very interesting and thought full for scholars and also leading factor for other researchers.

Authors' Response: We sincerely thank the reviewer for the encouraging feedback and for recognizing the contribution of this study.

Saying this, I have seen the title "Delays in seeking abortion and its determinants among reproductive-age women based on the Ethiopian Demographic and health survey"
I do have some clarification about this manuscript and your research work?

1. In line 101: Various barriers continue to prevent women from seeking timely abortion care. These barriers include "restrictive legal frameworks", how the restrictive legal framework is was barrier for delay for seeking abortion?

Authors' Response: Thank you for raising this important point. Restrictive legal frameworks can delay abortion care by limiting the conditions under which abortion is legally permitted, requiring complex procedures, and creating uncertainty or lack of awareness among women about their rights. These factors may discourage women from seeking services promptly, thereby contributing to delays. We have clarified this issue in the revised manuscript (lines 102 - 104).

2. Why you used 8 machine learning models and which problem is solved by using those models as machine learning researcher why not taking 4 or 5 models enough?

Authors' Response: Thank you for this thoughtful comment. We included eight machine learning models to ensure robust comparison across diverse algorithms with different learning strategies (linear, tree-based, distance-based, and probabilistic). Using multiple models helped us identify the most accurate and reliable model (Random Forest) for predicting delays and to validate that our findings were not dependent on a single modeling approach. We have clarified this in the revised manuscript (lines 224 - 232). We hope this addresses your concern, but we are happy to provide additional clarification if needed.

3. You and your team checks" Shapley Additive Explanations" in your research work, so which pattern or feature is more important and how do you measure.

Authors' Response: We appreciate the reviewer's comment. SHAP values were used to quantify each feature's contribution to predicting delays in seeking abortion, as fully described in the Methods section (lines 309 - 323). Briefly, the analysis showed that age (women aged 40–49), regional factors (residing in the Somali and Amhara regions), and lack of media exposure were the strongest positive contributors to delays, while urban residence and living in Addis Ababa were associated with lower likelihood of delay. These findings are clearly visualized in Figures 5 and 6, which display both beeswarm and bar plots for Random Forest and XGB models. We hope this provides a clear explanation of how feature importance was measured, and we are happy to provide further details if needed.

4. Did you use SHAP for measure the performance of deferent models if yes how and no why? Generally I have got the research work is important and thought full. But the document is better to upload in word document to address comments easily. Thank you

Authors' Response: We appreciate the reviewer's feedback and kind words. SHAP was not used to measure the performance of the different models; it was employed solely to interpret the contributions of individual features to the model predictions. Model performance was evaluated using standard metrics such as accuracy, AUC, sensitivity, and specificity, as described in the Methods and Results sections. To clarify this point, we have added a statement in the Methods section (Lines 309 - 311) noting that SHAP was used exclusively for feature interpretation and not for performance assessment.

Regarding the manuscript format, we acknowledge the suggestion and will ensure that a Word version is provided for ease of addressing reviewer comments. We are happy to provide any additional clarifications if needed.

Reviewer #2:

This study identifies key factors influencing delays in seeking abortion services in Ethiopia. The authors of this work claimed that the study offers valuable insights into the socio-demographic factors driving delayed access to abortion services by women aged 15-49 and these results carry important implications for both public health interventions and policy development in Ethiopia. Particularly, the findings offer valuable insights for designing public health initiatives aimed at reducing unsafe abortion-related maternal morbidity and mortality. However, I suggest the authors to address following concerns in order to enhance the quality of this manuscript.

1. How feature selections were carried out? In this issue, is there any support from previous work or how the authors have validated the feature selection criteria? Please mention the process evidence-based it way.

Authors' Response: Thank you for pointing this out. Feature selection was carried out using the Boruta algorithm, a method that identifies all relevant predictors by comparing original features to randomly permuted shadow features; only those consistently outperforming shadows are confirmed as important. This approach is supported by previous studies in health data science, and

epidemiologic studies [1, 2, 3]. In response to your comment and for transparency, we have included the feature selection plot in Supplementary Figure 1. We have also clarified this process in the revised Methods section (lines 192 -195) and are happy to provide further details if required.

2. Why eight machine learning algorithms were selected? Please justify.

Authors' Response: We thank the reviewer for the comment. As previously noted in response to Reviewer 1 (lines 224 - 232), we employed eight machine learning algorithms to ensure robustness and cover different model families, including linear, probabilistic, distance-based, and ensemble tree-based methods. This allowed us to compare performance across multiple paradigms, reduce model-specific bias, and identify the model that best captured the complexity of the data. We would be happy to provide further clarifications if needed or receive further suggestion from your side.

3. Please describe more about data set and its characteristics in the manuscript.

Authors' Response: We thank the reviewer for this important comment. A detailed description of the dataset and its characteristics was already included in the Results section under the sub-heading 'Sociodemographic characteristics'. In response to your comment and improve clarity and accessibility, we have now also added a description of the dataset in the Methods section (lines 161 to 166). Additionally, Supplementary Table 1 provides the full distribution of the sociodemographic features.

4. Please present some qualitative results as the behavior of a pregnant woman couldn't be determined by quantitative aspects only.

Authors' Response: Thank you for this insightful comment. However, the current study was based on secondary survey data, which only included quantitative variables. As such, qualitative aspects such as personal experiences, cultural influences, and contextual factors could not be directly captured. We agree that integrating qualitative approaches would enrich the understanding of women's health-seeking behavior, and we have highlighted this as a future research direction in the Conclusion and Recommendation section (Lines 607 - 610). We welcome any further suggestions or clarifications the reviewer may have on how best to integrate this perspective.

5.How this work is novel in terms of data curation, proposed methodology, optimizing existing algorithm and so on?

Authors' Response: We thank the reviewer for raising this important point. In response, we have revised the Introduction (Lines 127 - 135) to more clearly highlight the novelty of our work. Specifically, we emphasize that our study uniquely applies interpretable machine learning to the context of abortion care in Ethiopia, a rarely studied area. We also detail the novelty in terms of dataset curation (focusing on EDHS 2016 for abortion care), methodological contributions (feature selection via Boruta, model optimization, and SHAP-based explainability), and the balance achieved between predictive performance and interpretability. These revisions underline how our work advances beyond existing studies and provides actionable insights for maternal health policy.

6. Please interpret your result with explainable AI and also show the learning curve of the method.

Authors' Response: We thank the reviewer for this suggestion. We have interpreted our model results using explainable AI techniques, specifically Shapley Additive Explanations (SHAP), which quantify each feature's contribution to the model's predictions. The SHAP beeswarm and bar plots (Figures 5 and 6) clearly show the most influential features and their direction of effect. In addition, we have generated learning curves for the best performing model, i.e., Random Forest, to illustrate model performance and convergence with increasing training data, which we have included in Supplementary Figure 2 together with explanation in the revised manuscript (Lines 410 to 412). These additions enhance interpretability and provide insight into the robustness of our predictive models.

In summary, the topic of this study and the issue are relevant. But, the contribution of the authors are limited to:

a. using existing dataset,

b. applying machine learning algorithms on them, and

c. suggested some recommendations.

Authors' Response: Thank you for pointing this out. While the study uses an existing dataset, its contribution goes beyond data analysis by applying interpretable machine learning techniques (Boruta for feature selection, SHAP for explainability), optimizing multiple algorithms, and identifying key predictors of delayed abortion seeking. These findings provide actionable insights

to guide targeted public health interventions and policy decisions. We also acknowledge limitations, including reliance on self-reported data, potential recall and social desirability biases, and the absence of contextual qualitative insights, which we have discussed in the manuscript (lines 568 - 579). We welcome further suggestions on how to better highlight the study's value and address its limitations.

Please show the novelty of this research. This study is also missing with a new framework to handle the identified issues.

Authors' Response: We sincerely thank the reviewer for this observation. This comment overlaps with an earlier one, and we have already addressed it by revising the Introduction (Lines 127 - 135) to highlight the novelty of our work. Specifically, this study is the first to apply interpretable machine learning to abortion care delays in Ethiopia using nationally representative EDHS 2016 data. The novelty lies in combining rigorous feature selection (Boruta), model optimization through cross-validation, and SHAP-based explainable AI, providing a transparent framework for both prediction and interpretation. While our study focuses on methodological demonstration and policy-relevant insights, we also note that future work could extend this framework to design actionable interventions addressing the identified issues. We remain open to further questions or suggestions regarding the novelty and framework of our research.

References

1. Kursa MB, Jankowski A, Rudnicki WR. Boruta–a system for feature selection. Fundamenta informaticae. 2010 Jul;101(4):271-85.
2. Bajaj S, Behera L. Predictive Modeling of Cardiovascular Disease using Machine Learning Techniques. In2023 Third International Conference on Secure Cyber Computing and Communication (ICSCCC) 2023 May 26 (pp. 518-523). IEEE.
3. Zhou H, Xin Y, Li S. A diabetes prediction model based on Boruta feature selection and ensemble learning. BMC bioinformatics. 2023 Jun 1;24(1):224.
