# Supplementary material for: Interpretable machine learning for predicting delays in seeking abortion among reproductive-aged women in Ethiopia: A study using EDHS 2016 data
Source: PLOS Digit Health. 2026 Mar 13;5(3):e0001288. doi: 10.1371/journal.pdig.0001288 (PMC12987417; doi:10.1371/journal.pdig.0001288)
Supplement: Attachment — Submitted filename: revivew respo.docx [file pdig.0001288.s002.docx]

Thank you to look over my insight full expertise on this research work entitled “Interpretable Machine Learning for Predicting Delays in Seeking Abortion Among Reproductive-Aged Women in Ethiopia: : A Study Using EDHS 2016 Data” and I got this article is very interesting and thought full and identifying deferent Hedin factors that why mothers delay in Seeking Abortion service.

The research work is very interesting and thought full for scholars and also leading factor for other researchers

# **Saying this, I have seen the title** “Delays in seeking abortion and its determinants among reproductive-age women based on the Ethiopian Demographic and health survey”

I do have some clarification about this manuscript and your research work?

1. In line 101: Various barriers continue to prevent women from seeking timely abortion care. These barriers include “restrictive legal frameworks”, how the restrictive legal framework is was barrier for delay for seeking abortion
2. Why you used 8 machine learning models and which problem is solved by using those models as machine learning researcher why not taking 4 or 5 models enough?
3. You and your team checks” Shapley Additive Explanations” in your research work, so which pattern or feature is more important and how do you measure.
