# Supplementary material for: Interpretable machine learning for predicting delays in seeking abortion among reproductive-aged women in Ethiopia: A study using EDHS 2016 data
Source: PLOS Digit Health. 2026 Mar 13;5(3):e0001288. doi: 10.1371/journal.pdig.0001288 (PMC12987417; doi:10.1371/journal.pdig.0001288)
Supplement: Attachment — Submitted filename: Response to Reviewers_R2.docx [file pdig.0001288.s004.docx]

**Point by Point responses: ‘*Interpretable Machine Learning for Predicting Delays in Seeking Abortion Among Reproductive-Aged Women in Ethiopia: A Study Using EDHS 2016 Data*’ [Revision Two]**

We thank the Editor and the reviewers for their constructive comments and valuable suggestions. Many of the requested items were already included in Revision 1. Nevertheless, to enhance clarity and facilitate the assessment, we have made the relevant components more explicit, provided additional explanations and citations where appropriate, and added clear cross-referencing throughout the Methods, Results, and Discussion sections. In addition, we carefully reviewed the manuscript in its entirety to ensure consistency, accuracy, and clarity of presentation. We believe that these revisions have strengthened the manuscript and improved its overall readability. All changes are highlighted in the revised manuscript.

**Additional Editor Comments:**

The reviewer requests greater methodological clarity and justification throughout the manuscript. Specifically:

Feature Selection: Explain how features were selected, supported by evidence or prior studies, and describe the validation process used.

**Authors’ Response:** Thank you for this insightful comment. We have revised the Data Preprocessing section to provide a clearer rationale and methodological justification for the feature-selection process. Specifically, we now (i) explain why Boruta was chosen as the primary feature‑selection algorithm, (ii) support the selection of features using evidence from prior studies on determinants of delays in seeking reproductive health services, and (iii) describe the internal validation process used to confirm the stability and robustness of the selected features (lines 210 to 233).

We also have added the feature‑selection stability analysis we had conducted using repeated subsampling. The Boruta algorithm was run on 30 random 80% subsamples of the training data, and we now calculated the retention rate for each feature (i.e., the proportion of subsamples where Boruta classified it as Confirmed or Rejected). Features retained in ≥80% of iterations were considered stable, following stability‑selection recommendations. A stability plot summarizing these results has been included in the Supplementary Figure 2.

Algorithm Choice: Justify the selection of the eight machine learning algorithms applied in the study.

**Authors’ Response:** Thank you for pointing this out. We have now expanded the justification for selecting the eight machine‑learning algorithms. Specifically, the revised text clarifies that the algorithms were chosen to represent diverse modeling paradigms (linear, probabilistic, distance‑based, and ensemble tree‑based), ensuring comprehensive comparison, reducing model‑specific bias, and capturing different types of relationships in the data. We also added rationale grounded in typical applications of each algorithm in health and epidemiological prediction studies. The amended text appears in the Methods section (lines 266 to 299).

Dataset Description: Provide a more detailed description of the dataset, including its characteristics and relevant attributes.

**Authors’ Response:** Thank you very much for this helpful comment. We agree that providing a fuller description of the dataset strengthens the clarity and reproducibility of the study. In response, we have expanded the ‘Dataset and Study Variables’ section to more clearly describe the key characteristics of the dataset, including sociodemographic, reproductive, household, and behavioral attributes. We now explain the types of variables included, their categorical structure, and their relevance to the analysis. The revised section is provided in the revised manuscript on lines 161 to 184.

Qualitative Insights: Include qualitative analysis or discussion to complement the quantitative findings, especially considering that behavioral aspects (e.g., of pregnant women) may not be fully captured numerically.

**Authors’ Response:** Thank you for this thoughtful and constructive comment. We agree that behavioral and socio‑cultural aspects influencing delayed abortion seeking may not be fully captured through quantitative variables alone. To address this, we have expanded the Discussion section by adding a qualitative‑oriented insight that synthesizes existing contextual knowledge about women’s lived experiences, decision‑making processes, and socio‑cultural dynamics surrounding abortion in Ethiopia. This addition complements the quantitative machine‑learning findings and provides a more nuanced interpretation of the behavioral mechanisms underlying delayed abortion seeking. The newly added paragraph with relevant citations is presented in the Discussion section (lines 591 to 603).

Novelty and Contribution: Clearly articulate the novelty of the work—whether in data curation, methodological innovation, or algorithmic optimization.

**Authors’ Response:** Thank you for pointing this out. We have revised the manuscript to clearly articulate what makes the work original, including (1) the application of interpretable machine learning methods to a nationally representative dataset on abortion-seeking delays in Ethiopia, (2) the integration of the latest and robust AI explainability analysis (i.e., SHAP-based interpretability) to uncover behavioral and structural determinants, and (3) the methodological innovation of using a model-comparison framework to identify best-performing predictive algorithms in this context. A new paragraph emphasizing these contributions has now been added to the Discussion section on lines 604 to 618.

Explainability and Learning Curves: Interpret results using explainable AI techniques and present the learning curves for the proposed models.

We look forward to reading your revised paper.

**Authors’ Response:** Thank you for pointing this out. We would like to respectfully note that we have already applied the most robust and latest explainable AI techniques using SHAP values to interpret the results of the two top-performing models, Random Forest and XGBoost (lines 448 to 526). The SHAP bar and Beeswarm plots (Figures 4-6) provide detailed insights into feature importance and the direction and magnitude of feature contributions to model predictions, highlighting key factors such as age, regional differences, and media exposure. Please also note that we would be pleased to consider and respond to any specific suggestions from the editor and/or reviewers.

In addition, in response to the other important suggestion, we have now generated and consolidated learning curves for all eight models (RF, GBM, LightGBM, XGBoost, SVM, Logistic Regression, KNN, and Naive Bayes), illustrating how model accuracy evolves with increasing training data. These learning curves provide a clear view of model stability, generalization, and performance across varying training set sizes (Supplementary Figure 3). We hope these additions address the concern and we remain open to further suggestions to improve both the clarity and interpretability.
